# Large-scale behavioral characterization of oxycodone self-administration in heterogeneous stock rats reveals initial analgesic effects are associated with addiction-like behaviors

**DOI:** 10.1038/s41386-026-02348-8

**Published:** 2026-01-30

**Authors:** Marsida Kallupi, Giordano de Guglielmo, Lieselot L. G. Carrette, Sierra Simpson, Jenni Kononoff, Adam Kimbrough, Lauren C. Smith, Kokila Shankar, Alicia Avelar, Dana Conlisk, Molly Brennan, Lani Tieu, Sharona Sedighim, Brent Boomhower, Lisa Maturin, McKenzie J. Fannon, Angelica Martinez, Caitlin Crook, Selen Dirik, Nathan Velarde, Paul Schweitzer, Selene Bonnet-Zahedi, Elizabeth Sneddon, Sonja Plasil, Alex A. Morgan, Dyar N. Othman, Benjamin Sichel, Beverly Peng, Apurva S. Chitre, Oksana Polesskaya, Justin Lau, Ashley Vang, Leah C. Solberg Woods, Abraham A. Palmer, Olivier George

**Affiliations:** 1 Department of Psychiatry, University of California San Diego, La Jolla, CA USA; 2 Department of Neuroscience, The Scripps Research Institute, La Jolla, CA USA; 3 Section on Molecular Medicine, Department of Internal Medicine, Wake Forest University School of Medicine, Winston-Salem, NC USA; 4 Institute for Genomic Medicine, University of California San Diego, La Jolla, CA USA

**Keywords:** Reward, Motivation

## Abstract

Family and twin studies indicate that 20–60% of the vulnerability to opioid use disorder (OUD) is influenced by genetic factors, but the specific genes driving addiction-like behaviors, including sensitivity to opioid analgesia, tolerance, dependence, and escalation of oxycodone self-administration, remain unidentified, limiting precision medicine approaches. To address this, we phenotyped over 500 heterogeneous stock (HS) rats, an outbred population with high genetic diversity, to characterize traits associated with OUD vulnerability and resilience. Rats self-administered oxycodone (150 µg/kg/infusion) in short-access (2 h/day, 4 days) followed by long-access (12 h/day, 14 days) sessions. We assessed motivation for oxycodone using progressive ratio testing, withdrawal-induced hyperalgesia with von Frey tests, and tolerance to oxycodone’s analgesic effects via tail immersion tests. Large cohorts (*n* = 46–60) and *Z*-score normalization minimized cohort-specific effects. An Addiction Index, derived from averaging *Z*-scores of escalation, motivation, tolerance, and hyperalgesia, revealed significant individual variability. Rats with severe addiction-like behaviors displayed higher initial analgesia, greater escalation, and more pronounced tolerance compared to resilient rats. Females showed increased escalation and motivation compared to males, but similar tolerance and hyperalgesia. Principal component analysis confirmed the Addiction Index’s validity, accounting for 40% of behavioral variance. This high-throughput phenotyping in HS rats, leveraging their genetic diversity, provides a robust framework for genome-wide association studies to identify gene variants linked to OUD vulnerability, offering translational potential for discovering novel therapeutic targets and advancing pharmacogenetic strategies for OUD treatment.

## Introduction

The opioid crisis remains a pressing public health challenge, with over 2 million Americans affected by opioid use disorder [1]. Over the past 15 years, oxycodone consumption has surged by ~500%, paralleled by a quadrupling of opioid-related overdose deaths [2, 3]. While opioids effectively manage acute and chronic pain for some patients [4], their addiction risk complicates clinical decision-making, as little is known about how pain mechanisms interact with prescription opioids to drive addiction vulnerability.

Twin studies have estimated the heritability of OUD from 20 to 60% [5–7]. Human genome-wide association studies (GWAS) have identified loci associated with addiction-related traits [8–14], and more recent work has specifically examined OUD and related traits [15–20]. Despite this progress, the genetic and biological mechanisms mediating individual differences in opioid effects and addiction-like behaviors are poorly defined [21, 22].

Advances in preclinical GWAS methodologies have overcome previous limitations, such as insufficient recombination, by using outbred populations like heterogeneous stock (HS) rats, which are a genetically diverse outbred population created by interbreeding eight inbred strains that have undergone extensive recombination to enable fine-scale genetic mapping [23, 24]. Preclinical models offer standardized, longitudinal, and quantitative behavioral assessments in controlled environments, alongside opportunities for genetic manipulation.

A key advancement in preclinical opioid research is the extended-access oxycodone self-administration model, which exhibits high face, predictive, and construct validity for OUD [22, 25–27]. Unlike human studies, where GWAS for substance-specific OUD are lacking, preclinical models can identify novel genetic targets for specific substances, such as heroin [28–30] or oxycodone. Here, we used the extended-access oxycodone self-administration model to phenotype >500 HS rats. Rats were assessed for escalation, motivation, analgesia, tolerance, and withdrawal-induced hyperalgesia, integrated into an Addiction Index [31] to distinguish vulnerable and resilient individuals. This approach lays a robust foundation for GWAS to uncover genetic variants driving oxycodone addiction-like behaviors.

## Methods

Detailed procedures can be found in the George lab protocol repository on protocols.io (https://www.protocols.io/view/oxycodone-iv-self-administration-14egnze2pg5d/v1).

### Animals

HS rats (Rat Genome Database NMcwiWFsm #13673907, *n* = 542), developed at the NIH in the 1980s to maximize genetic diversity [32], were provided by Dr. Leah Solberg Woods (Medical College of Wisconsin, now at Wake Forest University School of Medicine). HS rats were derived from interbreeding eight inbred rat strains (ACI/N, BN/SsN, BUF/N, F344/N, M520/N, MR/N, WKY/N, and WN/N) and maintained with a large number of breeder pairs using a randomized breeding strategy, with each pair contributing one male and one female per generation to minimize inbreeding and genetic drift [24]. Each rat was implanted with an RFID chip. Rats were shipped at 3–4 weeks of age, quarantined for 2 weeks, and housed in pairs in a temperature- (20–22 °C) and humidity-controlled (45–55%) vivarium with a 12 h/12 h reversed light/dark cycle. They had ad libitum access to tap water and food pellets (PJ Noyes Company, Lancaster, NH, USA). A total of 700 HS rats were initially used in the intravenous oxycodone self-administration study. Complete and usable data were ultimately obtained from 542 animals, corresponding to an attrition rate of 22.6% (158 rats). Animals were excluded for one or more of the following reasons: loss of catheter patency or inconsistent drug delivery, post-surgical complications (including implantation failures, infections or sepsis requiring euthanasia on animal-welfare grounds), hardware or infusion-system malfunctions, severe opioid-induced respiratory depression (overdose), general health deterioration (≥20% body-weight loss, marked lethargy, or other signs of distress necessitating euthanasia), missing, corrupted, or incomplete experimental records. All procedures adhered to the National Institutes of Health Guide for the Care and Use of Laboratory Animals and were approved by the Institutional Animal Care and Use Committees of The Scripps Research Institute and University of California, San Diego.

### Drugs

Oxycodone hydrochloride (National Institute on Drug Abuse, Bethesda, MD, USA) was dissolved in 0.9% sterile saline and administered intravenously at 150 μg/kg/infusion (free base, 0.1 ml volume over 6 s). This dose was selected based on previous studies demonstrating reliable self-administration and escalation in rats [31, 33, 34], as well as its ability to achieve plasma concentrations (~40 ng/ml) associated with pharmacological effects [35]. To ensure that all rats received the same dose of oxycodone in mg/kg despite the fixed infusion duration, the concentration of oxycodone was individually adjusted in each syringe according to the animal’s body weight.

### Intravenous catheterization

Rats were anesthetized with vaporized isoflurane (1–5%) and implanted with intravenous catheters in the right jugular vein using established procedures described previously [31]. Catheters consisted of 18 cm Micro-Renathane tubing (0.023-inch inner diameter, 0.037-inch outer diameter; Braintree Scientific, Braintree, MA, USA) connected to a 90°-angled guide cannula (Plastics One, Roanoke, VA, USA), secured in dental acrylic and anchored with a 2 cm diameter, 1 mm thick mesh. Tubing was inserted into the vein following a needle puncture (22 G) and secured with a suture. The guide cannula was punctured through a small incision on the back. The cannula exited through a small dorsal incision and was sealed with a plastic cap and metal cover to ensure sterility and protection. Post-surgery, rats received flunixin (2.5 mg/kg, s.c.) for analgesia and cefazolin (330 mg/kg, i.m.) for infection prophylaxis. Catheters were flushed daily with heparinized saline (10 U/ml of heparin sodium; American Pharmaceutical Partners, Schaumberg, IL, USA) in 0.9% bacteriostatic sodium chloride (Hospira, Lake Forest, IL, USA) containing cefazolin (52.4 mg/0.2 ml). Rats recovered for 7 days before self-administration, with daily monitoring to ensure catheter patency and animal welfare.

### Behavioral testing

#### Operant self-administration

Oxycodone self-administration (SA) was conducted in operant conditioning chambers (29 × 24 × 19.5 cm; Med Associates, St. Albans, VT, USA) housed within sound-attenuating, ventilated cubicles. Chambers were constructed of transparent plastic front and back walls, opaque metal side walls, two retractable levers, and a cue light on the front panel. Sessions began with lever extension. Responses on the right (active) lever delivered oxycodone (150 μg/kg/infusion in 0.9% saline, 0.1 ml over 6 s) via an infusion pump connected to intravenous catheters, following a fixed-ratio 1 schedule. Each infusion was followed by a 20-s timeout, signaled by a cue light above the active lever, during which additional presses were recorded but had no effect. Responses on the left (inactive) lever were recorded without consequences. Fluid delivery and behavioral data were controlled using MED-PC IV software (Med Associates). Rats were initially trained in four short-access (ShA) sessions (2 h/day) to establish stable self-administration. Subsequently, they underwent 14 long-access (LgA) sessions (12 h/day, Monday–Friday) to assess escalation of oxycodone intake, starting at the onset of the dark phase of the 12 h/12 h light/dark cycle. Food and water were available ad libitum during LgA sessions, and a fresh wooden block (3 × 3 × 3 cm) was provided daily to enrich the self-administration chambers.

#### Progressive ratio testing

**Fig. 1:**
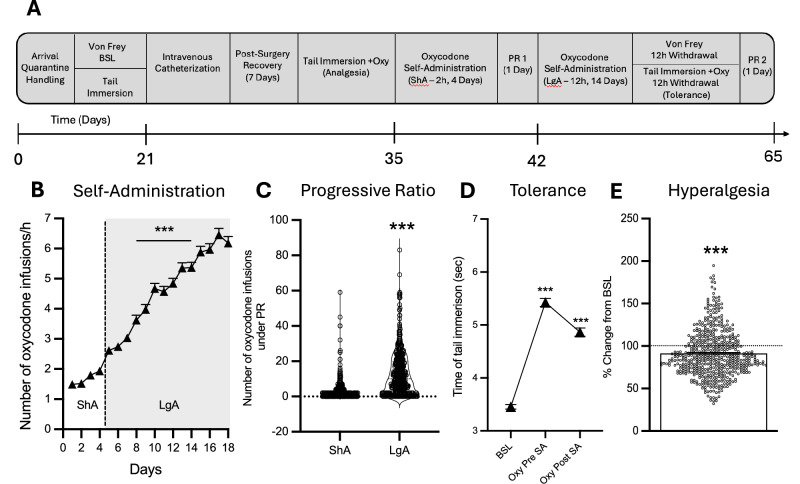
Individual differences in addiction-behaviors in HS rats following intravenous oxycodone self-administration. **A** Timeline of the behavioral paradigms. **B** Number of oxycodone infusions (150 μg/kg/infusion) during the first hour of self-administration under ShA (2 h/day), and LgA (12 h/day) conditions across 14 cohorts (*N* = 542). Data show significant escalation of intake from day 4 of LgA onward (***p < 0.001 vs. first LgA session, one-way ANOVA with Bonferroni post hoc comparisons). **C** Violin plot depicting the number of oxycodone infusions during PR testing after ShA and LgA phases (*N* = 542, ***p < 0.001, paired *t*-test). **D** Development of tolerance to oxycodone’s analgesic effects. Tail withdrawal thresholds (in seconds) measured via the tail immersion test at baseline (BSL), after three oxycodone infusions pre-extended-access self-administration (Oxy-pre-SA), and post-extended-access self-administration (Oxy post-SA) (***p < 0.001 vs BSL and ###*p* < 0.001 vs Oxy-pre-SA, one-way ANOVA with Bonferroni post hoc comparisons). **E** Development of withdrawal-induced hyperalgesia. Data represent percentage change from baseline in paw withdrawal force (g) measured via the von Frey test before and after extended-access oxycodone self-administration (***p < 0.001 vs BSL, paired *t*-test).

Rats were tested on a progressive ratio (PR) schedule of reinforcement at the end of each phase (once after ShA and once after LgA, see Fig. 1 for a detailed timeline). In the PR paradigm, the number of lever presses required for each oxycodone infusion (150 µg/kg/infusion) increased progressively within each session as follows: 1, 1, 2, 2, 3, 3, 4, 4, 6, 6, 8, 8, 10, 11, 12, 13, 14, 15, 16, 17, etc +1 until 50, 60, 70, 80, 90, 100. The breakpoint was defined as the last completed ratio before a 60-min period without completing the next ratio, terminating the session [36].

#### Mechanical nociceptive von Frey testing

Withdrawal-induced hyperalgesia was assessed using a dynamic plantar aesthesiometer (Ugo Basile, Gemonio, Italy) adapted from established protocols [37–39]. Mechanical sensitivity was measured by applying a linearly increasing force to the plantar surface of each hind paw until withdrawal occurred. The force (in grams) and latency to paw withdrawal were recorded, with three measurements per paw averaged for each rat. Testing was conducted at two time points: (i) baseline (prior to oxycodone exposure) and (ii) during acute withdrawal, 10–12 h after the final long-access (LgA) oxycodone self-administration session (see Fig. 1A for timeline). Baseline data are expressed as force (g), while withdrawal data are reported as the percent change in force from each rat’s baseline to enable within-subject evaluation of hyperalgesia.

#### Analgesia by tail immersion testing

Rats were gently restrained in a soft cloth pocket, and the distal half of the tail was submerged in a 52 °C water bath. Latency to tail withdrawal was recorded, with a 10 s cut-off to prevent tissue damage. Two measurements, taken 30 s apart, were averaged for each rat. Testing occurred at three time points: (i) baseline (pre-drug exposure), (ii) prior to oxycodone ShA self-administration, 15 min after two intravenous infusions of oxycodone (150 μg/kg, 0.1 ml volume), and (iii) 2 h post the final long-access (LgA) session (day 14), 15 min after two identical oxycodone infusions. The post-LgA test evaluated tolerance to oxycodone’s analgesic effects. The 15 min post-infusion timing was chosen based on peak plasma oxycodone concentrations and the 12 h withdrawal period aligned with prior studies assessing opioid tolerance [40].

### Statistical analyses

All data were analyzed using Prism 9.0 (GraphPad, San Diego, CA, USA). Self-administration data, including escalation of oxycodone intake during short-access (ShA; 2 h/day) and long-access (LgA; 12 h/day) sessions, were analyzed using repeated-measures one-way analysis of variance (ANOVA) with session as the within-subject factor, followed by Bonferroni post hoc tests to compare oxycodone infusions across days. For sex differences in self-administration, a two-way repeated-measures ANOVA was used with sex as the between-subject factor and session as the within-subject factor, followed by Bonferroni post hoc tests. Progressive ratio (PR) data were analyzed using a two-way ANOVA with sex or group (resilient, mild, moderate, severe) as the between-subject factor and time (post-ShA vs. post-LgA) as the within-subject factor, followed by Bonferroni post hoc tests for pairwise comparisons. Tail immersion test data, assessing oxycodone’s analgesic effects and tolerance, were analyzed using a two-way ANOVA with group or sex as the between-subject factor and time (baseline, pre-ShA, post-LgA) as the within-subject factor, followed by Bonferroni post hoc tests. Von Frey test data, evaluating withdrawal-induced hyperalgesia, were analyzed using paired *t*-tests to compare baseline and post-LgA withdrawal thresholds within groups, and one-way ANOVA with Bonferroni post hoc tests for between-group comparisons. Pairwise comparisons between groups (e.g., resilient vs. severe) were conducted using unpaired Student’s t-tests. Pearson’s correlation analysis was used to examine relationships between behavioral measures (e.g., escalation, motivation, tolerance, hyperalgesia). To account for cohort variability, behavioral data from 14 cohorts (*n* = 46–60 per cohort) were normalized using *Z*-scores, calculated as *Z* = (*x*−*μ*)/*σ*, where *x* is the raw value, *μ* is the cohort mean, and σ is the cohort standard deviation. The Addiction Index was computed as the mean of *Z*-scores for escalation, PR breakpoint, tolerance, and hyperalgesia. Principal component analysis (PCA) was performed to validate the Addiction Index, retaining components with eigenvalues > 1, using R (version 4.2.1). Data are reported as mean ± SEM unless otherwise specified, with statistical significance set at *p* < 0.05. All tests were two-tailed, and exact p-values are provided where possible. More analyses are provided in the Supplementary Material (Figs. S1–S3).

## Results

### Evaluation of oxycodone addiction-like behaviors in HS rats

Addiction-like behaviors were assessed in 542 HS rats across 14 cohorts (*n* = 46–60 per cohort) using a standardized oxycodone self-administration protocol (Fig. 1A). Following jugular catheterization and a 7-day recovery, rats underwent four daily short-access (ShA; 2 h/day) sessions to establish oxycodone self-administration, followed by 14 daily long-access (LgA; 12 h/day) sessions to evaluate intake escalation. During the extended-access oxycodone self-administration phase, animals showed significant escalation of intake, as measured by the significant increase in oxycodone rewards per hour from day 4 of LgA onward compared to the first day of LgA (Fig. 1B): *F*_(17,8687)_ = 205.2, *p* < 0.0001, one-way ANOVA, with Bonferroni post hoc comparisons. Inactive lever responding remained very low (<15 responses/session in most animals) showing negligible inter-individual variability (Fig. S1). Motivation for oxycodone was assessed after both ShA and LgA phases using a progressive ratio (PR) test (Fig. 1C). The number of oxycodone infusions significantly increased after extended-access of oxycodone self-administration, compared to levels at the end of the short-access phase (*t*_541_ = 18.25, *p* < 0.001). Given that PR data exhibit a non-normal distribution characterized by a significant floor effect and right skew, these results are also presented on a logarithmic scale in Fig. S2 to visualize the distribution of lower-responding subjects. Rats were also tested for baseline pain response, oxycodone’s analgesic effects, and the development of tolerance to these effects using the tail immersion test at three different phases of the behavioral protocol (see Fig. 1A for a detailed testing timeline). As shown in Fig. 1D, HS rats developed tolerance to the analgesic effects of oxycodone following extended-access to oxycodone self-administration (*F*_(2,1076)_ = 279.4, *p* < 0.0001, one-way ANOVA). Bonferroni post hoc comparisons revealed a significant decline in oxycodone’s analgesic effects after 14 days of extended-access of oxycodone self-administration (*p* < 0.001, Fig. 1D). Finally, oxycodone withdrawal-induced hyperalgesia was assessed using the von Frey Test. Paw withdrawal responses to a mechanical stimulus were measured at the beginning and at the end of the behavioral paradigm (pre- and post- oxycodone, Fig. 1E), with data expressed as a percentage reduction in force (g) compared to baseline responses. Results demonstrated that HS rats developed withdrawal-induced hyperalgesia following extended-access oxycodone self-administration (*t*_541_ = 7.41, *p* < 0.001, Fig. 1E).

### Sex differences in oxycodone addiction-like behaviors in HS rats

**Fig. 2:**
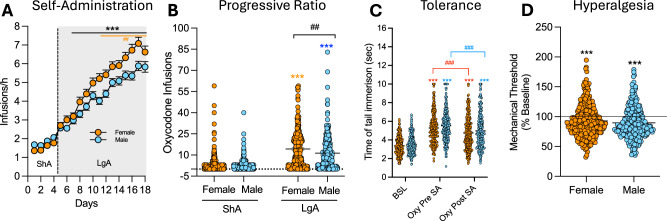
Sex differences in addiction-like behaviors. **A** Number of oxycodone infusions (150 μg/kg/infusion) during the first hour of short-access (ShA; 2 h/day) and long-access (LgA; 12 h/day) oxycodone self-administration in male and female HS rats (*N* = 542). Females exhibited greater escalation from day 6 of LgA onward (***p < 0.01 vs LgA 1 and ##*p* < 0.01 vs males, two-way ANOVA with Bonferroni post hoc tests). **B** Violin plot depicting the number of oxycodone infusions under progressive ratio (PR) testing after ShA and LgA phases in male and female HS rats (*N* = 542; ***p < 0.01 vs ShA, ##*p* < 0.01 vs males, two-way ANOVA with Bonferroni post hoc tests). **C** Development of tolerance to oxycodone’s analgesic effects. Tail withdrawal thresholds (in seconds) measured via the tail immersion test at baseline (BSL), after two oxycodone infusions pre-extended-access self-administration (Oxy-pre-SA), and post- extended-access self-administration (Oxy post-SA) in male and female HS rats (*N* = 542, ***p < 0.001 vs BSL and ###*p* < 0.001 vs Oxy-pre-SA, one-way ANOVA with Bonferroni post hoc tests). **D** Development of withdrawal-induced hyperalgesia in male and female HS rats. Data represent percent change from baseline in paw withdrawal force (g) measured via the von Frey test before and after extended-access oxycodone self-administration (*N* = 542; ***p < 0.001 vs BSL, paired *t*-test).

No sex differences were observed during the acquisition phase under ShA (2 h/day) conditions. However, during LgA (12 h/day), female HS rats exhibited greater escalation of oxycodone intake compared to males (Fig. 2A). The two-way ANOVA with sex as the between-subjects factor and sessions as the within-subject factor revealed significant main effects of sex (*F*_(__1542)_ = 6.577; *p* < 0.001), sessions (*F*_(17,9210)_ = 224.5; *p* < 0.001) and their interaction (*F*_(17,9210)_ = 5.661; *p* < 0.001). However, calculation of the effect size for the main effect of sex yielded a partial eta squared (*η*^2^_*p*_) of 0.012, indicating that while statistically significant, biological sex accounted for a small proportion (~1.2%) of the total variance in intake. Bonferroni post hoc test demonstrated that females self-administered significantly more oxycodone than males from day 6 of the LgA phase (day 10 in Fig. 2A) through the end of the behavioral protocol (*p* < 0.01). In the progressive ratio (PR) test, a two-way ANOVA with sex as the between-subject factor and time (ShA vs. LgA) as the within-subject factor showed a significant main effect of sex (*F*_(1540)_ = 8.28; *p* < 0.01), time (*F*_(1540)_ = 337.2; *p* < 0.001), and their interaction (*F*_(1,540)_ = 4.83; *p* < 0.05). Like intake, the effect size for sex on motivation was small (*η*^2^_*p*_ = 0.015), suggesting that sex differences explain approximately 1.5% of the phenotypic variance. Bonferroni post hoc tests confirmed that motivation for oxycodone increased in both sexes from ShA to LgA (*p* < 0.001), with females displaying significantly higher motivation than males after LgA (*p* < 0.01, Figs. 2B and 5B). Male and female HS rats showed comparable baseline response in the tail immersion test, similar responses to oxycodone’s analgesic effects, and equivalent development of tolerance to these effects, with no significant treatment x sex interaction (*F*_(2,1074)_ = 0.19; *p* = NS, Fig. 2C). Similarly, no sex differences were observed in the development of withdrawal-induced hyperalgesia, as assessed by paw withdrawal thresholds, in the von Frey test during withdrawal (*t*_540_ = 1.32; *p* = NS, Fig. 2D).

### Addiction Index: evaluation of individual differences in addiction-like behaviors

**Fig. 3:**
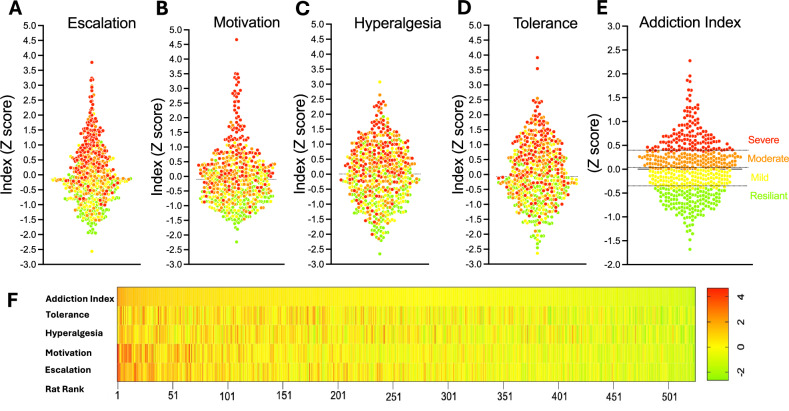
Construction and visualization of the Addiction Index for oxycodone addiction-like behaviors. **A**–**D** *Z*-scores for individual measures in the HS rat population (*n* = 542). **A** Escalation of oxycodone intake (average of last 3 days of long-access [LgA] self-administration), **B** motivation (progressive ratio [PR] breakpoint post-LgA, **C** withdrawal-induced hyperalgesia (percent reduction in von Frey test thresholds), and **D** tolerance (difference in tail immersion test responses pre- and post-LgA). **E** Addiction Index, calculated as the mean of the four *Z*-scores, categorizing rats into quartiles: severe (red), moderate (orange), mild (yellow), and resilient (green). The scatter plot depicts individual rats along the principal analysis, highlighting resilient (green) and severe (red) groups. **F** Addiction Index distribution for individual rats, showing constituent *Z*-scores and classifications by vulnerability (resilient vs. severe) and sex (male vs. female).

To quantify individual variability in oxycodone addiction-like behaviors, we used an Addiction Index (Fig. 3E) [31, 41–45], integrating four measures: escalation of oxycodone intake under a fixed-ratio (FR) schedule of reinforcement, motivation under a progressive ratio (PR) schedule, tolerance to oxycodone’s analgesic effects, and withdrawal-induced hyperalgesia. Each measure was normalized within cohorts (*n* = 46–60) and sex using *Z*-scores. To validate the necessity of this approach, we analyzed the raw, non-normalized total oxycodone intake across cohorts (Fig. S3). A one-way ANOVA revealed significant inter-cohort variability (*F*_(11,540)_ = 3.254; *p* = 0.0003), confirming the presence of batch effects. Consequently, within-cohort normalization was utilized to remove these technical confounds, ensuring that the subsequent stratification reflected intrinsic biological phenotype rather than experimental batch variation.

*Z*-scores were calculated as (*Z* = $\frac{x-\mu }{\sigma }$), where $\chi$ is the raw value, $\mu$ is the mean of the cohort per sex, and $\sigma$ is the standard deviation of the cohort per sex. We thus obtained an Escalation Index (Fig. 3A), PR Index (Fig. 3B), Tolerance Index (Fig. 3C), and Hyperalgesia Index (Fig. 3D). To quantify the intensification of drug taking relative to initial exposure, daily intake values were normalized against the distribution of the first day LgA phase. Specifically, the *Z*-score for any given session *i* (*Z*_*i*_) was calculated using the individual’s daily intake (*X*_i_) relative to the group mean (*μ*_day1_) and standard deviation (*σ*_day1_) of the first day of escalation, according to the formula: *Z*_*i*_ = (*X*_*i*_−*μ*_day1_)/*σ*_day1_. The final Escalation Index for each subject was defined as the average of these *Z*-scores calculated over the final three sessions of the self-administration phase. The PR index used the *Z*-score post-LgA breakpoint. The tolerance index was calculated from the *Z*-score of the difference in tail immersion test response pre- and post- LgA. The Pain Index reflected the *Z*-score of the percent reduction in von Frey test withdrawal thresholds during withdrawal relative to baseline. The overall Addiction Index was computed as the mean of these four *Z*-scores (Fig. 3E). PCA of the four normalized measures identified a first principal component (PC1) with an eigenvalue > 1 that explained ~40% of the total variance, with component loadings ranging from *r* = 0.19 to 0.86. Detailed examination revealed that PC1 was heavily dominated by self-administration behaviors (escalation: *r* = 0.86; motivation: *r* = 0.86), while physiological measures contributed minimally to this component (hyperalgesia: *r* = 0.19; tolerance: *r* = 0.19). Consistent with this, the correlation matrix confirmed a moderate association between intake and motivation (*r* = 0.53) but low correlations between behavioral and physiological measures (*r* < 0.10). Consequently, utilizing a scoring system based on PC1 weights would result in an index that disproportionately reflects drug-taking behavior while effectively excluding physiological dependence. To avoid this bias and ensure the phenotype reflects a comprehensive addiction syndrome encompassing both drug-seeking and physical dependence (consistent with clinical diagnostic criteria), we rejected the PC1-weighted approach and retained the equal-weighting strategy (simple average of the four *Z*-scores) for the Addiction Index. Rats were ranked by Addiction Index and divided into four equal quartiles- resilient, mild, moderate, and severe-to classify vulnerability (Fig. 3F).

**Fig. 4:**
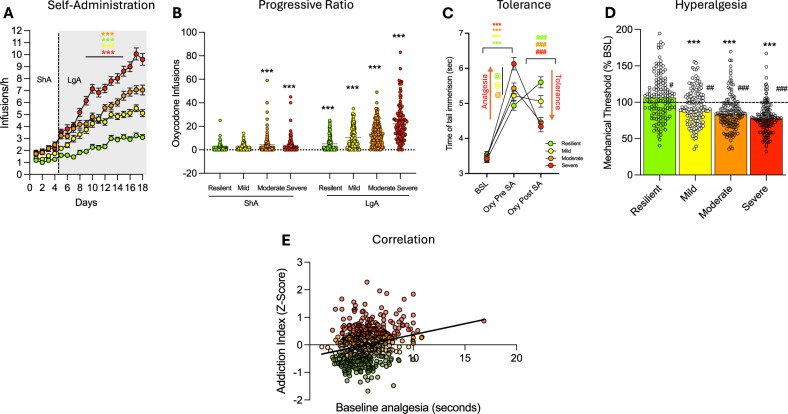
Differential vulnerability to oxycodone addiction-like behaviors in HS rats. **A** Oxycodone infusions during short-access (ShA; 2 h/day) and long-access (LgA:12/day) self-administration in resilient, mild, moderate, and severe groups (***p < 0.001). **B** Motivation assessed by progressive ratio (PR) testing post- ShA and post-LgA, showing infusions per session (***p < 0.001 vs ShA, #*p* < 0.05 and ###*p* < 0.001 vs immediate lower group). **C** Tolerance to oxycodone’s analgesic effects measured by tail immersion test at baseline (BSL), pre-ShA (15 min post-oxycodone, 150 μg/kg/infusion × 2), and post- LgA (12 h withdrawal, post-oxycodone). Data show tail withdrawal (***p < 0.001 vs BSL and ###*p* < 0.001 vs Oxy-pre-SA, @*p* < 0.05 vs immediate lower group). **D** Withdrawal-induced hyperalgesia assessed by von Frey test, expressed as percent change in paw withdrawal force from baseline at 10–12 h post-LgA (***p < 0.001 vs BSL; #*p* < 0.05, ##*p* < 0.01, and ###*p* < 0.001 vs. immediate lower group). **E** Linear regression analysis correlating baseline oxycodone-induced analgesia (tail immersion) with the final Addiction Index for all subjects (*n* = 542). Each dot represents an individual animal. The analysis reveals a highly significant positive correlation (*r* = 0.2426, *p* < 0.0001), identifying high initial physiological sensitivity as a risk factor for the development of the severe addiction phenotype.

**Fig. 5:**
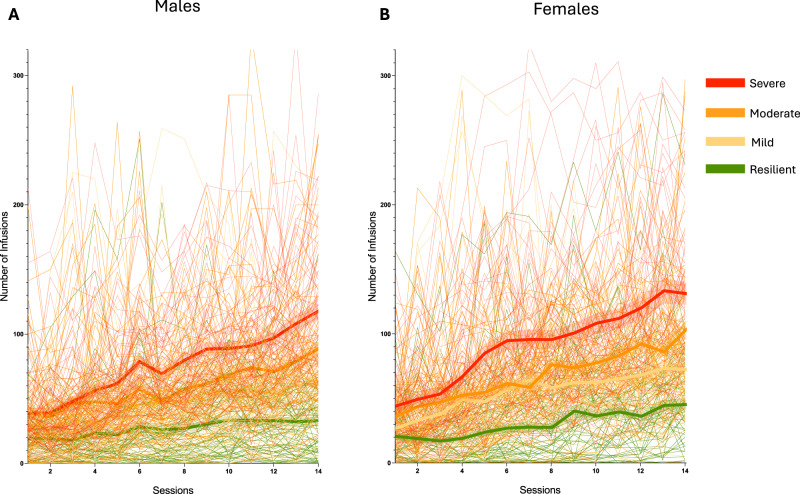
Individual drug self-administration trajectories reveal distinct cluster-specific acquisition patterns across sexes. Longitudinal oxycodone self-administration data for **A** males and **B** females. Thin semi-transparent lines represent the daily infusion counts for individual rats, color-coded by their designated cluster (red = *severe*, orange = *moderate*, yellow = *mild*, green = *resilient*). Thick solid lines with shaded bands represent the Mean ± SEM for each cluster. The visualization highlights the extensive individual variability within the heterogeneous stock (HS) population and confirms that the identified clusters capture distinct acquisition trajectories that are masked by the total population average.

Oxycodone intake increased across Addiction Index-defined groups (resilient, mild, moderate, severe). Two-way ANOVA (group x session) revealed significant effects of group (*F*_(3, 538)_ = 75.85; *p* < 0.0001), session (*F*_(17, 538)_ = 239.1; *p* < 0.0001), and their interaction (*F*_(51, 9095)_ = 18.48; *p* < 0.0001). To quantify the magnitude of this phenotypic stratification independent of sample size, we calculated the effect size, yielding a partial eta squared (*η*^2^_*p*_) of 0.30, indicating a large effect. Pairwise comparisons confirmed that each group self-administered significantly more oxycodone than its immediate lower group (resilient < mild < moderate < severe, Fig. 4A). To fully characterize the behavioral diversity inherent in the HS rats, we examined individual acquisition trajectories across the self-administration phase. While the population mean suggests a steady escalation of intake, determining individual performance reveals substantial heterogeneity. Figure 5 displays the daily infusion rates for every subject, stratified by sex and colored by cluster assignment. This granular view confirms that the identified clusters represent robust, distinct behavioral phenotypes. Specifically, “*severe*” animals (red) consistently exhibit rapid escalation of oxycodone intake, whereas “*resilient*” animals (green) maintain low, stable intake levels throughout the acquisition period, a pattern observed in both males (Fig. 5A) and females (Fig. 5B). In progressive ratio (PR) testing (Figs. 4B and S2C), two-way ANOVA (group × time) showed significant effects of group (*F*_(3, 538)_ = 60.04; *p* < 0.0001), time (*F*_(1, 538)_ = 404.3; *p* < 0.0001), and their interaction (*F*_(3, 538)_ = 38.7; *p* < 0.0001). To quantify the magnitude of the phenotypic differentiation, we calculated the effect size for the main effect of group, yielding a partial eta squared (*η*^2^_*p*_) of 0.25. This indicates a large biological effect, confirming that the identified clusters exhibit robust differences in motivational drive. Pairwise comparisons showed that all groups increased breakpoints from short-access (ShA) to long-access (LgA) phases (*p* < 0.01), with each group showing higher motivation than its immediate lower group (severe > moderate > mild > resilient; *p* < 0.05).

In the tail immersion test, two-way ANOVA (group x time) showed a significant interaction (*F*_(6, 1070)_ = 22.00; *p* < 0.0001). Bonferroni post hoc tests demonstrated all groups showed increased analgesia post-oxycodone infusion (150 μg/kg × 2) pre-ShA (*p* < 0.001 vs. baseline; Fig. 4C). The severe group showed greater analgesia than other groups (*p* < 0.001, Fig. 4C). To determine if initial sensitivity to the analgesic effects of oxycodone serves as a predictive biomarker for addiction vulnerability, we correlated baseline oxycodone-induced analgesia with the final Addiction Index (Fig. 4E). Linear regression analysis revealed a highly significant positive correlation (*r* = 0.2426, *P* < 0.0001). However, the coefficient of determination (*R*^2^ = 0.0589) indicates that analgesic sensitivity explains only a small fraction of the total phenotypic variance. After extended access to oxycodone self-administration, moderate and severe animals showed development of tolerance to the analgesic effects of oxycodone (as demonstrated by the decreased response in the tail immersion test after a dose of oxycodone compared to the response pre-self-administration). The magnitude of this physiological adaptation over time was substantial (*η*^2^_*p*_ = 0.37), confirming the robust induction of opioid tolerance in the high-intake groups, perhaps due to greater drug exposure. On the other hand, the resilient group showed a significantly increased response compared to the baseline (pre-self-administration), suggestive of a sensitization of the analgesic effect of oxycodone that was probably due to the low and intermittent level of oxycodone intake during extended access to oxycodone self-administration (Fig. 4C). This effect in the resilient group also extended to the von Frey test. The analysis showed that all the groups showed emergence of hyperalgesia during acute withdrawal from oxycodone (*p* < 0.001 vs BSL) except for the resilient group, which showed instead a significantly increased paw withdrawal response in the von Frey test after extended access to oxycodone self-administration (*p* < 0.05 vs BSL). The one-way ANOVA test showed a significant difference between the groups (*F*_(3, 538)_ = 30.64; *p* < 0.0001). Calculation of the effect size yielded an eta squared (*η*^2^_*p*_) of 0.15, indicating a large effect and confirming that withdrawal-induced hyperalgesia is strongly dependent on the addiction phenotype. The Bonferroni post hoc test demonstrated that each of the subgroups (divided based on their Addiction Index) showed increased oxycodone withdrawal-induced hyperalgesia compared to their immediate lower subgroup (resilient < mild < moderate < severe, Fig. 4D).

**Fig. 6:**
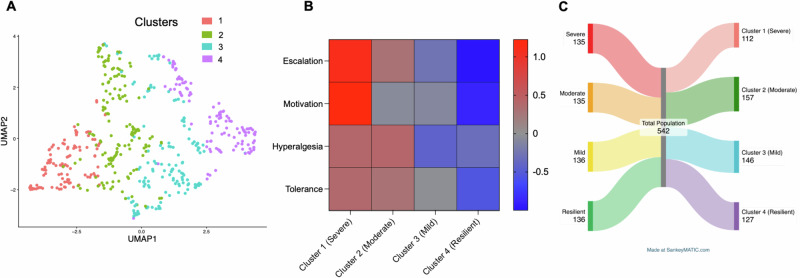
Unsupervised Bayesian Stochastic Block Model (BSBM) clustering validates the behavioral phenotypes. **A** Uniform Manifold Approximation and Projection (UMAP) plot visualizing the data structure of the total population (*n* = 542). Points are colored by the four clusters identified via the unsupervised BSBM algorithm. **B** Behavioral heatmap profiling the four unsupervised clusters. Rows represent *Z*-score normalized behavioral features; columns represent the clusters. The color scale indicates deviation from the population mean. The analysis confirms that *Cluster 1* aligns with the *severe* phenotype, while *Cluster 4* aligns with the *resilient* phenotype. **C** Sankey diagram illustrating the concordance between the original behavioral phenotypes (Left) and the unsupervised BSBM clusters (Right). The flow width represents the number of animals. A Chi-square test indicates a significant dependence between the manual and unsupervised classification methods (*χ*^2^ = 863.1, df = 9, *p* < 0.0001).

To confirm that our stratification strategy captures true biological variation rather than arbitrary cutoffs, we performed an unsupervised clustering analysis on the raw behavioral data. We utilized the Bayesian Stochastic Block Model (BSBM) algorithm, which constructs a subject-subject similarity network to infer latent population structures [29, 46]. This unbiased analysis identified four stable clusters (Fig. 6A). We profiled these clusters using a behavioral heatmap (Fig. 6B), which revealed that Cluster 1 displayed a “*severe*” behavioral profile (high susceptibility), whereas Cluster 4 displayed a “*resilient*” profile. A Sankey diagram (Fig. 6C) illustrates the correspondence between these unsupervised clusters and our originally defined groups. A Chi-square test confirmed a highly significant association between the two methods (*χ*^2^ = 863.1, df = 9, *p* < 0.0001), validating our stratification criteria.

## Discussion

We conducted a comprehensive behavioral screening of 542 genetically diverse HS rats to characterize oxycodone addiction-like behaviors, including escalation of intake, motivation, tolerance, and withdrawal-induced hyperalgesia. We employed a state-of-the-art model of extended access to intravenous self-administration [47, 48], which induces neuroadaptations observed in clinical populations [49–53], and recapitulates seven DSM-5 criteria for severe opioid use disorder: tolerance [54], withdrawal [55, 56], excessive intake [57], unsuccessful quitting efforts [58, 59], time spent obtaining the drug [60], reduced social/recreational activities [50, 61], and continued use despite adverse consequences [53, 62–64]. Escalation of oxycodone intake and increased PR responding are interpreted as compulsive use [26]. Unlike prior studies using smaller cohorts, inbred strains [65], or mouse panels (BXD [66], HMDP [67]) and proxies like locomotion [65] passive drug effects [68], this study leverages the genetic diversity of HS rats, which exhibit high inter-individual variability and low intra-individual variability in oxycodone self-administration, providing a robust platform for identifying specific behavioral phenotypes associated with compulsive oxycodone use.

While distinct addiction-related traits—escalation, motivation, and tolerance—are known to be independently heritable [69], the specific phenotypic architecture of oxycodone vulnerability has remained elusive. By computing a multidimensional Addiction Index, we stratified the population into resilient, mild, moderate, and severe quartiles. Resilient rats exhibited minimal escalation and low motivation, whereas “*severe*” rats displayed a robust addiction-like phenotype characterized by high intake, profound tolerance, and significant withdrawal-associated hyperalgesia

Our analysis identified a critical dissociation between physiological sensitivity and addiction vulnerability. Interestingly, the “*severe*” group displayed greater analgesic sensitivity to oxycodone prior to self-administration. However, this group also developed the most substantial tolerance post-escalation. Regression analysis revealed that while initial high analgesic sensitivity is a significant statistical predictor of future vulnerability (*p* < 0.0001), it explains only a small fraction of the variance (*R*^2^ ≈ 6%). This suggests that while initial physiological sensitivity is a risk factor, it is not a standalone fate; the development of the full addiction syndrome requires the recruitment of distinct motivational neurocircuitry.

Furthermore, we observed substantial heterogeneity in withdrawal-induced hyperalgesia. As revealed by our PCA, hyperalgesia loaded weakly on the primary addiction component (*r* = 0.19) compared to voluntary intake measures (*r* = 0.86). This indicates that in a genetically diverse population, the severity of physiological withdrawal is not strictly commensurate with the magnitude of drug intake. This uncoupling of physical and motivational symptoms validates our decision to use a composite Addiction Index rather than relying on any single metric. While alternative approaches such as Principal Component Regression might treat physiological adaptations solely as external predictors of intake, our “syndrome-based” model integrates tolerance and hyperalgesia as intrinsic components of the disorder, consistent with their status as core diagnostic criteria in the DSM-5.

Regarding sex differences, female HS rats self-administered significantly more oxycodone than males during the long-access phase, consistent with previous literature [33, 35]. Prior evidence suggests only mild sex differences in oxycodone pharmacokinetics (PK) [70]. However, more granular human studies indicate lower plasma concentrations in women due to distribution volume differences [71–73]. Consistent with this complexity, we recently characterized the four inbred founder strains of the HS rat and found that while sex differences in oxycodone pharmacokinetics were present, these peripheral PK profiles did not linearly predict susceptibility to self-administration [74]. Furthermore, recent work assessing a panel of inbred strains demonstrated that the emergence of sex differences in oxycodone self-administration is highly strain-dependent, reinforcing that the magnitude of this effect is modulated by genetic background [75]. This dissociation suggests that the elevated intake in female HS rats is not driven by simple metabolic deficits (e.g., faster clearance). Instead, it likely reflects behavioral compensation to overcome differences in brain oxycodone availability (as hypothesized in our previous work in Wistar rats [33]) or reduced mu-opioid receptor efficacy, necessitating higher intake to achieve comparable central reward magnitude.

Although most individuals initiate oxycodone use via the oral route, oral bioavailability of oxycodone in rats is extremely low (1.2–5.0%; [76]) compared to 60–87% in humans [77], which has led most rat studies to use the intravenous route to ensure reliable brain exposure. Nevertheless, recent oral oxycodone self-administration studies in rats have consistently shown robust intake, with females self-administering significantly more oxycodone than males [78–80]. The marked sex differences observed in these oral studies mirror the female-greater intake seen in the current intravenous paradigm, indicating that fundamental sex-dependent vulnerability mechanisms operate independently of administration route.

While ours is the first study to examine extended access *oxycodone* in HS rats at this scale, similar work using *heroin* identified three vulnerability clusters, confirming our observation that females are overrepresented in the vulnerable phenotype [28, 29]. Notably, this work extended stratification to the neural level, identifying distinct, sex-specific patterns of Fos activation distinguishing resilience from vulnerability [29]. Together, these studies across different opioids validate the HS rat model as a robust platform for capturing the individual variability necessary to dissect both the behavioral and neuronal mechanisms of addiction.

Finally, this study lays the foundation for future genetic analysis. Previous studies using classical inbred rat strains have established substantial heritability for multiple opioid-related traits, including baseline heroin self-administration [81–83], escalation of heroin intake [84], preferred opioid dose [84], acute reactivity to opioid withdrawal [85, 86], antinociceptive effect [87], opioid-induced conditioned place preference [88], proenkephalin levels [81], and m opioid receptor regulation [89]—findings that underscore the genetic underpinnings of opioid use disorder observed in humans. Building on this foundation, the HS rat population used here is optimized for fine-mapping these genetic loci [23, 24]. Ongoing studies are dissecting sex differences in the neurobiological adaptations produced by addiction-like behaviors to oxycodone [90–92], and future studies will integrate the behavioral phenotypes characterized here with genotype data to identify gene variants associated with vulnerability and resilience.

Additionally, tissue samples from these behaviorally and genetically characterized rats are available through the oxycodone biobank [43], facilitating the investigation of non-genetic mechanisms, such as epigenetic, transcriptomic [68], and microbiomic factors—that underlie the transition from controlled use to addiction.

## Supplementary information

Supplementary Information
