# Supplementary material for: Large-scale behavioral characterization of oxycodone self-administration in heterogeneous stock rats reveals initial analgesic effects are associated with addiction-like behaviors
Source: Neuropsychopharmacology. 2026 Jan 30;51(6):1074–83. doi: 10.1038/s41386-026-02348-8 (PMC13125645; doi:10.1038/s41386-026-02348-8)
Supplement: Supplementary file 1 [file 41386_2026_2348_MOESM1_ESM.docx]

**Supplementary Information:**

**Supplementary Figure 1. Assessment of Lever Discrimination Accuracy During Extended-Access Self-Administration.** Longitudinal analysis of active and inactive lever presses across the 14 daily sessions of long-access (LgA, 12 h/day) oxycodone self-administration. Data are presented as mean ± SEM for the total heterogeneous stock (HS) cohort (N=542). Animals exhibited robust discrimination between the drug-paired (Active) and non-paired (Inactive) levers throughout the experiment. Active lever responding (black line) escalated significantly over time, whereas inactive lever responding (grey line) remained stable and negligible (<15 responses/session).

**Supplementary Figure 2. Log-Transformed Progressive Ratio (PR) Performance Confirms Robust Group Differences.** Progressive ratio breakpoints are plotted on a logarithmic scale (Log10) to visualize the distribution of low-responding subjects and account for the exponential nature of the schedule. **(A)** Total population performance (n=542) during pre-escalation (Short Access) vs. post-escalation (Long Access) testing. **(B)** PR breakpoints stratified by sex (Males vs. Females) across timepoints. **(C)** PR breakpoints stratified by the four behavioral phenotypes (*severe, moderate, mild, resilient*). Data patterns on the log scale mirror those observed on the linear scale (Figure 1 and 2), confirming that the identification of the '*severe*' phenotype and the overall statistical conclusions are not artifacts of the data distribution.

**Supplementary Figure 3. Significant Inter-Cohort Variability in Raw Self-Administration Data Necessitates Within-Cohort Normalization.** Quantification of raw (non-normalized) total oxycodone infusions over the 14-day self-administration period across the 12 independent cohorts used in the study (n=542 total). Data are presented as box-and-whiskers plots: center line = median; box= interquartile range; whiskers = minimum to maximum.  A one-way ANOVA revealed significant differences in raw intake levels between cohorts (F(11,530)=3.254, p=0.0003). Post-hoc analysis (Tukey's test) confirmed systematic differences between specific cohorts (e.g., Cohorts 7 and 8 vs. Cohort 1, p<0.05), supporting the necessity of within-cohort Z-score normalization to ensure accurate phenotypic stratification independent of technical variation.
